# Short-Term Functional Outcomes in Patients Undergoing Primary Total Knee Arthroplasty According to Their Body Mass Index

**DOI:** 10.7759/cureus.101269

**Published:** 2026-01-10

**Authors:** Ranj Bhakar, Arjun S Chakrapani, Arfaz Shaik, Aaron Alexander, Thivagar Murugesan, Prasanna Kumar Anbazhagan, Dan Ghent

**Affiliations:** 1 Department of Trauma and Orthopaedics, Torbay Hospital, Torquay, GBR; 2 Department of Orthopaedics and Trauma, Croydon University Hospital NHS Trust, London, GBR; 3 Department of Trauma and Orthopaedics, Wrightington Wigan and Leigh NHS Trust, Wigan, GBR; 4 Department of Orthopaedics, University Hospital Llandough, Penarth, GBR; 5 Department of Trauma and Orthopaedics, Royal Shrewsbury Hospital, Shrewsbury, GBR; 6 Department of General Surgery, Ashford and St Peter's Hospital, Chertsey, GBR; 7 Department of Internal Medicine, Queen's Medical Centre, Nottingham, GBR

**Keywords:** body mass index, complications, knee society score, length of stay, patient-reported outcomes, total knee arthroplasty

## Abstract

Objective: This study aimed to evaluate the association between body mass index (BMI) and short-term outcomes following primary unilateral total knee arthroplasty (TKA), including functional improvement, perioperative variables, and postoperative complications.

Methodology: A retrospective case-control study was conducted at a tertiary orthopedic center on 525 consecutive patients who underwent primary unilateral TKA for osteoarthritis between January 2019 and December 2023. Patients were classified according to World Health Organization (WHO) BMI criteria: normal (18.5-24.9 kg/m²), overweight (25.0-29.9 kg/m²), and obese (≥30.0 kg/m²). Collected data included demographics, American Society of Anesthesiologists (ASA) grade, Charlson Comorbidity Index (CCI), glycated hemoglobin (HbA1c), operative time, length of hospital stay, change in Knee Society Score (ΔKSS) at 12 months, patient-reported outcome measures, satisfaction, revision surgery, and postoperative complications. Between-group comparisons were performed using one-way analysis of variance (ANOVA) for continuous variables and chi-square or Fisher’s exact tests for categorical variables, with significance set at p < 0.05.

Results: Baseline characteristics were comparable across BMI groups, except for higher ASA scores and HbA1c levels in obese patients (p < 0.05). Obese patients had significantly longer operative times (95.9 ± 16.8 minutes) and hospital stays (4.8 ± 1.2 days) than normal-weight patients (83.6 ± 14.1 minutes; 3.9 ± 1.0 days, p < 0.01). All BMI groups demonstrated significant improvement in ΔKSS at 12 months, although the gain was lowest in obese patients (34.7 ± 10.5 vs. 41.2 ± 9.6; p = 0.012). Overall complications were highest in the obese group with 16 patients (9.1%), followed by nine patients (5.1%) in the overweight group and seven patients (4.0%) in the normal-weight group, with wound-related issues being the most common. Revision surgery occurred in one (0.6%) obese patient, and no mortality was reported.

Conclusion: Higher BMI was associated with longer operative times, prolonged hospital stays, increased wound complications, and slightly reduced functional improvement and satisfaction at 12 months after primary TKA. These findings highlight the importance of optimizing metabolic status before surgery and emphasize the need for individualized perioperative risk assessment in patients undergoing knee arthroplasty.

## Introduction

Total knee arthroplasty (TKA) is a widely performed and effective procedure for end-stage knee osteoarthritis, capable of significantly reducing pain and improving function in most patients. With the growing global volume of TKA procedures, particularly within high-volume healthcare systems, there is increasing interest in identifying patient-related factors that influence perioperative risk and postoperative recovery [1,2].

Body mass index (BMI) is among the most extensively studied modifiable patient factors in TKA research due to its potential association with surgical complexity, complication risk, implant survivorship, and functional outcomes. Several large cohort studies have reported higher rates of wound complications, prosthetic joint infection, and early revision among patients with elevated BMI; however, findings regarding functional improvement and patient-reported satisfaction after TKA remain inconsistent [3-5]. Although obesity is recognized as an independent risk factor, variability in functional outcomes highlights the need for careful patient selection, counseling, and perioperative optimization [6,7].

Despite growing evidence, uncertainties persist regarding clinically meaningful BMI thresholds, the extent to which BMI independently predicts risk after adjusting for comorbidities and metabolic variables such as glycemic control (HbA1c), and whether observed differences in functional improvement across BMI categories are clinically significant. These knowledge gaps complicate individualized decision-making and contribute to variability in institutional criteria for surgical eligibility and preoperative optimization protocols [8-10].

Therefore, this study aimed to evaluate the association between BMI category and short-term outcomes following primary unilateral TKA at a high-volume tertiary care center. Specifically, it assessed the 12-month change in Knee Society Score (ΔKSS) as the primary outcome, and perioperative parameters, such as length of stay, operative time, complication rates (overall and wound-related), revision surgery within 12 months, and patient satisfaction, as secondary outcomes.

## Materials and methods

Study design and population

This retrospective case-control study included consecutive adult patients aged 18-85 years who underwent primary unilateral TKA for osteoarthritis at a high-volume tertiary orthopedic center. Clinical, surgical, and postoperative data were retrieved from the institutional electronic medical record system, and patient-reported outcome measure (PROM) data were obtained from a prospectively maintained outcomes database to ensure accuracy and completeness. Only patients with complete perioperative information and at least 12 months of follow-up were included. Institutional Review Board approval was obtained before initiating the study, and all procedures adhered to the ethical principles of the Declaration of Helsinki.

Sample size

The sample size was calculated a priori using G*Power software (version 3.1, Universität Düsseldorf, Germany) [11]. Assuming a moderate effect size (Cohen’s d = 0.5), an alpha of 0.05, 80% power, and equal allocation across the three BMI groups, a minimum of 158 patients per group was required. For continuous outcomes, the sample size estimation followed the standard formula for comparing means across multiple groups:

\[ n = \frac{2\sigma^{2}(Z_{\alpha/2} + Z_{\beta})^{2}}{\Delta^{2}} \]

where $\sigma$ is the estimated standard deviation, $\Delta$ is the minimum clinically relevant difference between groups, $Z_{\alpha/2} = 1.96$ for a two-tailed $\alpha = 0.05$, and $Z_{\beta} = 0.84$ corresponding to 80\% power.

To account for potential data loss, missing data, or incomplete 12-month PROM follow-up, the target sample size was increased to 175 patients per BMI category, yielding a total planned sample of 525 participants. BMI groups were defined according to World Health Organization (WHO) criteria: normal weight (18.5-24.9 kg/m²), overweight (25.0-29.9 kg/m²), and obese (≥30.0 kg/m²) [12].

Selection criteria

Eligible participants were adults aged 18-85 years who underwent primary unilateral TKA for radiographically and clinically confirmed osteoarthritis. Inclusion required documented baseline BMI, available PROM data, and verified 12-month postoperative follow-up. Exclusion criteria included revision TKA, staged or simultaneous bilateral TKA, and inflammatory arthropathies such as rheumatoid or psoriatic arthritis. Patients with active infections at surgery, cognitive impairment limiting reliable PROM completion, or incomplete postoperative outcome data were also excluded.

Data collection and outcomes

Data extraction followed a standardized protocol. Demographic and clinical variables included age, sex, American Society of Anesthesiologists (ASA) grade, Charlson Comorbidity Index (CCI), and preoperative HbA1c. Perioperative variables, including operative time and length of hospital stay (LOS), were obtained from surgical and inpatient records. Patient-reported and functional outcomes were evaluated using the KSS and satisfaction questionnaires [13]. These measures enabled calculation of 12-month changes in KSS (ΔKSS) and determination of clinically meaningful improvement based on established thresholds. The primary outcome was the 12-month ΔKSS, selected because it reflects both functional recovery and patient-perceived improvement after TKA, and the 12-month time point is widely accepted as representing a stable postoperative outcome following completion of rehabilitation. Additionally, ΔKSS accounts for baseline functional differences across BMI groups, enabling a more meaningful comparison of postoperative improvement rather than relying solely on absolute postoperative scores. Postoperative complications within 12 months, including wound problems, infections requiring reoperation, thromboembolic events, major adverse events, and revision procedures, were identified through the electronic medical record. Wound-related complications were defined as postoperative wound issues, including superficial wound dehiscence, delayed wound healing, persistent wound drainage, and superficial surgical site infection not requiring deep debridement or implant removal.

Statistical analysis

Continuous variables were reported as mean ± standard deviation (SD) for normally distributed data or as median with interquartile range (IQR) for non-normally distributed data. Normality was assessed using the Shapiro-Wilk test. Group comparisons were performed using one-way ANOVA with Bonferroni post-hoc testing for normally distributed variables, or the Kruskal-Wallis test for non-parametric variables. Categorical variables were compared using the chi-square test or Fisher’s exact test, as appropriate. All analyses were conducted using IBM SPSS Statistics for Windows, Version 29 (Released 2023; IBM Corp., Armonk, New York, United States).

## Results

A total of 525 patients were included in the study, with 175 (33.3%) patients in each BMI group. The mean age of participants was 67.2 ± 8.3 years, and 320 (61.0%) were female. Baseline characteristics were comparable across the three BMI groups, except for significantly higher ASA classifications and preoperative HbA1c levels in the obese participants (Table 1).

**Table 1:** Baseline demographic and clinical characteristics Data have been presented as number (%) for categorical variables and as mean ± standard deviation for continuous variables. Between-group comparisons were performed using one-way analysis of variance (ANOVA) for continuous variables and the chi-square test for categorical variables, with statistical significance set at p < 0.05 * indicates a significant p-value

Variable	Normal (n=175) (kg/m²)	Overweight (n=175) (kg/m²)	Obese (n=175) (kg/m²)	Test value	p-value
Age (years)	66.8 ± 8.1	67.5 ± 8.4	67.4 ± 8.5	0.33	0.72
Female (N; %)	106 (60.6%)	104 (59.4%)	110 (63.4%)	1.25	0.54
American Society of Anesthesiologists Score (ASA, units)	2.1 ± 0.5	2.2 ± 0.5	2.4 ± 0.6	4.01	0.02*
HbA1c (%)	5.8 ± 0.7	6.0 ± 0.8	6.3 ± 0.9	4.55	0.018*
Charlson comorbidity index (CCI, units)	2.3 ± 1.2	2.4 ± 1.3	2.5 ± 1.4	0.89	0.41

Surgical duration and LOS increased progressively with higher BMI. All BMI groups demonstrated significant improvement in KSS at 12 months; however, the magnitude of improvement was lower in the obese participants. The proportion of patients achieving clinically meaningful improvement in PROMs decreased with increasing BMI: 160 (91.4%) in the normal-weight group, 156 (89.1%) in the overweight group, and 146 (83.4%) in the obese group. Similarly, patient-reported satisfaction declined with higher BMI (Table 2).

**Table 2:** Perioperative and functional outcomes Data have been presented as number (%) for categorical variables and as mean ± standard deviation for continuous variables. Between-group comparisons were performed using one-way analysis of variance (ANOVA) for continuous variables and the chi-square test for categorical variables, with statistical significance set at p < 0.05 * indicates a significant p-value

Outcome	Normal (kg/m²)	Overweight (kg/m²)	Obese (kg/m²)	Test values	p-value
Operative time (minutes)	83.6 ± 14.1	88.2 ± 15.3	95.9 ± 16.8	12.8	<0.001*
Length of stay (in days)	3.9 ± 1.0	4.2 ± 1.1	4.8 ± 1.2	9.5	<0.01*
ΔKnee Society Score at 12 months	41.2 ± 9.6	39.8 ± 10.1	34.7 ± 10.5	5.2	0.012*
PROM improvement	155 (88.4%)	152 (86.9%)	144 (82.1%)	4.7	0.09
Patient satisfaction	160 (91.4%)	156 (89.1%)	146 (83.4%)	6.1	0.04*

The overall complication rate was 32/525 (6.1%), with the highest incidence in the obese group. Wound complications occurred in 5/175 (2.9%) normal-weight, 6/175 (3.4%) overweight, and 12/175 (6.9%) obese patients (p = 0.031). Infections were reported in 1/175 (0.6%), 2/175 (1.1%), and 3/175 (1.7%) patients, respectively (p = 0.27). Thromboembolic events occurred in 1/175 (0.6%) patients in each group (p = 1.00). Total complications were observed in 7/175 (4.0%) normal-weight, 9/175 (5.1%) overweight, and 16/175 (9.1%) obese patients (p = 0.04). Revision surgery within 12 months was required in 1/175 (0.6%) obese patients, with none in the other groups (p = 0.32). No mortality occurred during follow-up (Table 3).

**Table 3:** Postoperative complications (*) shows a significant p-value

Complication	Normal (kg/m²)	Overweight (kg/m²)	Obese (kg/m²)	Chi-square test value	p-value
Wound complication	5 (2.9%)	6 (3.4%)	12 (6.9%)	6.8	0.031*
Infection	1 (0.6%)	2 (1.1%)	3 (1.7%)	2.6	0.27
Thromboembolism	1 (0.6%)	1 (0.6%)	1 (0.6%)	0.0	1.00
Total complications	7 (4.0%)	9 (5.1%)	16 (9.1%)	6.1	0.04*
Revision surgery	0 (0%)	0 (0%)	1 (0.6)	2.3	0.32

## Discussion

The outcomes of TKA are influenced by several patient-related factors, among which BMI remains of consistent clinical interest due to its potential impact on perioperative complexity, wound healing, and functional recovery. In the present study, an increasing BMI was associated with longer operative times, extended hospital stays, and higher rates of wound complications, as well as slightly lower functional improvement at 12 months. These findings are consistent with the existing literature, which indicates that obesity introduces both technical and metabolic challenges in joint arthroplasty [14].

A recent randomized controlled trial by Lobato et al. [12] demonstrated that navigation-assisted surgery (NAS) significantly improved mechanical alignment accuracy to within ±3° among obese patients undergoing TKA. Specifically, 69% of NAS patients achieved the desired precision compared to 47% in the conventional group (OR 2.5, p = 0.006). Although the navigation group had a modestly longer operative time (70 vs. 59 minutes), no significant differences were observed in hospital stay or complication rates. The study provides additional evidence that NAS enhances coronal tibial alignment accuracy in obese patients, who are inherently at a higher surgical risk, and significantly reduces the proportion of tibial alignment outliers (16% vs. 32%, p = 0.026) [12].

In alignment with these findings, our study showed that obese patients experienced significantly longer operative durations (95.9 ± 16.8 minutes) and hospital stays (4.8 ± 1.2 days) compared with normal-weight patients (83.6 ± 14.1 minutes; 3.9 ± 1.0 days). Although navigation-assisted techniques were not evaluated in the present analysis, the observed increase in operative time parallels the trends described by Lobato et al. [12] and Boyce et al. [15], supporting the notion that higher BMI is associated with greater surgical complexity and perioperative burden.

Regarding postoperative functional outcomes, improvement in the KSS was significantly lower in obese patients (ΔKSS = 34.7 ± 10.5) compared with normal-weight patients (41.2 ± 9.6; p = 0.012). While Lobato et al. [12] reported comparable functional outcomes at 12 months between navigation-assisted and conventional TKA in obese patients, several extensive registry-based studies have similarly found modest reductions in patient-reported outcomes with increasing BMI. These findings indicate that although obese patients clearly benefit from TKA, the magnitude of functional improvement is slightly attenuated compared with patients of normal weight. This difference may be partly attributed to biomechanical overload and chronic inflammatory processes associated with obesity [16,17].

The complication profile observed in this study aligns with prior meta-analyses linking obesity to increased wound-related morbidity following TKA. The wound complication rate among obese patients (6.9%) was more than twice that observed in normal-weight individuals (2.9%), consistent with previous evidence associating elevated BMI with a higher risk of superficial infection and delayed wound healing [14,18-20]. Importantly, the incidence of major complications, thromboembolic events, and revision surgery did not differ significantly between groups, suggesting that modern perioperative optimization strategies and stringent infection-control protocols may effectively mitigate severe adverse events in high-BMI populations.

Strengths and limitations

This study has several notable strengths, including a large sample size, a comprehensive assessment of both objective functional scores and patient-reported outcomes, and the incorporation of relevant metabolic parameters such as HbA1c. The single-center design also ensured consistency in surgical technique and perioperative management.

However, certain limitations should be acknowledged. The retrospective design introduces inherent selection and information biases, and the one-year follow-up limits insight into longer-term outcomes such as implant survival and late complications. Moreover, BMI is an imperfect surrogate for body composition, as it does not distinguish between fat and lean mass. Finally, the study’s generalizability may be limited by institutional practices and demographic characteristics specific to the population studied.

## Conclusions

In conclusion, a higher BMI is associated with longer operative duration, prolonged hospital stay, increased wound-related complications, and slightly reduced functional improvement and patient satisfaction following primary unilateral TKA. Nevertheless, all BMI groups derive substantial benefit from surgery, supporting the continued use of TKA in patients with obesity, provided that appropriate preoperative optimization and individualized risk counseling are undertaken. Future studies should focus on long-term functional and prosthetic outcomes, as well as refining perioperative management strategies to improve further results.
